# Placement on COVID-19 Units Does Not Increase Seroconversion Rate of Pediatric Graduate Medical Residents

**DOI:** 10.3389/fped.2021.633082

**Published:** 2021-04-29

**Authors:** Timothy Crisci, Samuel Arregui, Jorge Canas, Jenaya Hooks, Melvin Chan, Cory Powers, Andrew L. Schwaderer, David S. Hains, Michelle C. Starr

**Affiliations:** ^1^Medicine-Pediatric Residency, Indiana University, Indianapolis, IN, United States; ^2^Division of Nephrology, Department of Pediatrics, Indiana University, Indianapolis, IN, United States

**Keywords:** coronavirus disease 2019, graduate medical education, nosocomial spread, severe acute respiratory syndrome coronavirus 2, transmission

## Abstract

**Background:** Severe acute respiratory syndrome coronavirus 2 (SARS-CoV-2) and its associated disease COVID-19 (coronavirus disease 2019) has presented graduate medical education (GME) training programs with a unique set of challenges. One of the most pressing is how should hospital systems that rely on graduate medical residents provide appropriate care for patients while protecting trainees. This question is of particular concern as healthcare workers are at high risk of SARS-CoV-2 exposure.

**Objective:** This cross-sectional study sought to assess the impact of hospital COVID-19 patient placement on pediatric graduate medical residents by comparing rates of SARS-CoV-2 seroconversion rates of residents who worked on designated COVID-19 teams and those who did not.

**Methods:** Forty-four pediatric and medicine–pediatric residents at Riley Children's Hospital (Indianapolis, IN) were tested for SARS-CoV-2 immunoglobulin M (IgM) and IgG seroconversion in May 2020 using enzyme-linked immunosorbent assays (Abnova catalog no. KA5826), 2 months after the first known COVID-19 case in Indiana. These residents were divided into two groups: those residents who worked on designated COVID-19 teams, and those who did not. Groups were compared using χ^2^ or Fisher exact test for categorical variables, and continuous variables were compared using Student *t* testing.

**Results:** Forty-four of 104 eligible residents participated in this study. Despite high rates of seroconversion, there was no difference in the risk of SARS-CoV-2 seroconversion between residents who worked on designated COVID-19 teams (26% or 8/31) and those who did not (31% or 4/13). Eleven of 44 residents (25%) tested positive for SARS-CoV-2 IgG, whereas only 5/44 (11.4%) tested positive for SARS-CoV-2 IgM, without a detectable difference between exposure groups.

**Conclusion:** We did not observe a difference in SARS-CoV-2 seroconversion between different exposure groups. These data are consistent with growing evidence supporting the efficacy of personal protective equipment. Further population-based research on the role of children in transmitting the SARS-CoV-2 virus is needed to allow for a more evidence-based approach toward managing the COVID-19 pandemic.

## Background

Healthcare settings are at high risk for severe acute respiratory syndrome coronavirus 2 (SARS-CoV-2) exposure and spread (1, 2). SARS-CoV-2 and its associated disease, coronavirus disease 2019 (COVID-19), has presented graduate medical education (GME) training programs with a unique set of challenges. One of the most pressing is how should hospital systems that rely on graduate medical residents provide appropriate care for patients while simultaneously protecting their trainees (3, 4). This question is of particular concern given data suggesting that healthcare workers are at high risk of exposure to SARS-CoV-2 (5, 6).

Indiana emerged as a US COVID-19 hotspot in March 2020, with sustained ongoing community transmission and high rates of infection. On March 24, 2020, Riley Children's Hospital (Indianapolis, IN) created dedicated COVID-19 pediatric medicine teams and floors to limit SARS-CoV-2 exposure to trainees and other healthcare workers. Admitted patients tested for SARS-CoV-2 (including both suspected and confirmed cases) were admitted to these specific teams or units to limit healthcare worker exposure and nosocomial transmission. Patients admitted to COVID-19 teams had SARS-CoV-2 testing performed by nasopharyngeal polymerase chain reaction (PCR). Patients admitted without suspicion of SARS-CoV-2 did not have PCRs obtained. To further limit SARS-CoV-2 transmission, all healthcare workers at Riley Children's Hospital were required to wear surgical masks while in the hospital starting on March 25, 2020.

The objective of this study was to assess the impact of hospital COVID-19 patient placement on pediatric graduate medical residents by comparing rates of SARS-CoV-2 seroconversion rates of residents who worked on designated COVID-19 teams and those who did not. We hypothesized that (1) the rates of SARS-CoV-2 seroconversion would be high in pediatric graduate medical residents and that (2) those who worked on the designated COVID-19 teams would be more likely to have SARS-CoV-2 immunoglobulin M (IgM) or IgG antibodies.

## Methods

### Setting and Participants

All graduate medical residents in pediatrics, including pediatric, internal medicine/pediatric, and emergency medicine/pediatric residents working at Riley Children's Health, a 247-bed pediatric tertiary care hospital, between February 1, 2020, and May 22, 2020, were eligible for inclusion in this cross-sectional observational study. Residents were contacted by email and consented to participate. There were no exclusion criteria. Participating residents completed a brief survey that included signs and symptoms that could be attributable to SARS-CoV-2 infection (Supplementary Material).

### SARS-CoV2 Exposure Classification

Residents were divided into two groups based on SARS-CoV-2 exposure: those who had worked on a COVID-19 team or floor and those who had not. COVID-19 teams and floors included the emergency department (ED), the pediatric intensive care unit (PICU), the infectious disease service, and the dedicated COVID pediatric hospital medicine team, which was responsible for patients with suspected and confirmed SARS-CoV-2 infection. During the time period of this study (March–May 2020), there were ~20 to 25 COVID-19 patients taken care of by COVID-19 teams each week, making up ~10% of the inpatient total patient volume. Residents on COVID-19 teams wore N-95 masks, gowns, and eye protection for all patient encounters, and residents on non-COVID-19 teams wore surgical masks for all patient encounters. There were no shortages impacting access to personal protective equipment (PPE), and all trainees received both pre-COVID-19 training in proper use of PPE as well as just-in-time training during the COVID-19 pandemic in donning and doffing to ensure correct procedures. All participants provided consent to participate in the research study under Indiana University Institutional Review Board protocol no. 2003962066A008. All clinical investigations were conducted according to the principles expressed in the Declaration of Helsinki.

### Ascertainment of SARS-CoV-2 Antibody Status

**Figure 1:**
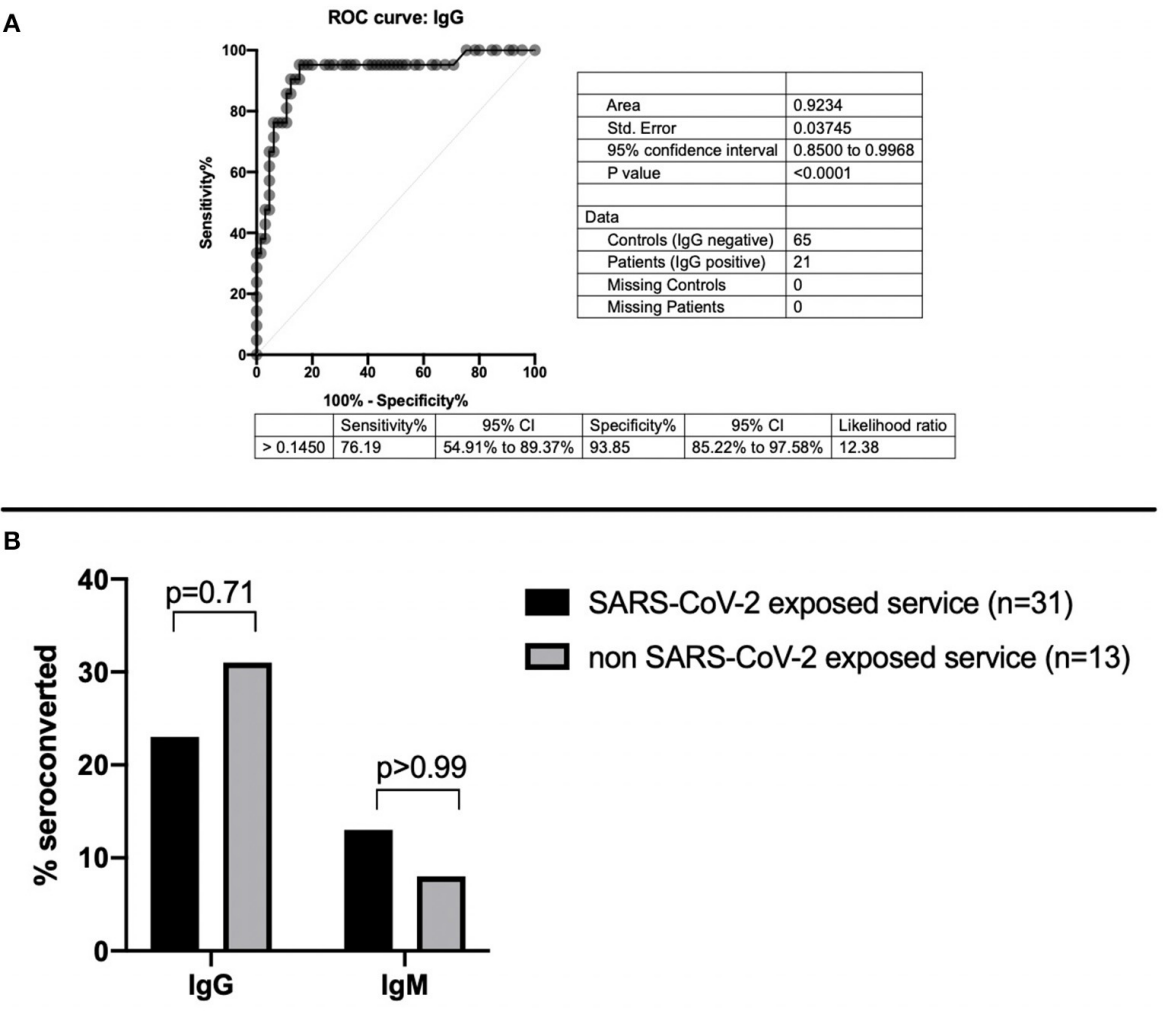
**(A)** Sensitivity and specificity of SARS-CoV-2 IgG ELISA. **(B)** SARS-CoV-2 IgG and IgM seroconversion based on exposure to COVID-19 patients.

In each participant, 5 mL of blood was obtained by direct venipuncture. Samples were collected between May 18, 2020, and May 22, 2020. Residents were tested for SARS-CoV-2 IgM and IgG antibodies using enzyme-linked immunosorbent assays (ELISA, Abnova catalog no. KA5826). Manufacturer's assay protocols were followed for the commercial ELISAs and previously published methods for our custom ELISAs (7, 8). As previously reported, the threshold for a positive ELISA test was set using 65 pre-COVID-19 pandemic samples collected in the 2011 calendar year by the Indiana Biobank and 21 samples from patients with documented positive SARS-CoV-2 nasopharyngeal PCR samples provided by the Eli Lilly Corporation (8). IgM threshold level with an optic density (OD) of 0.140 was used as previously reported (2). The reported sensitivity and specificity for IgM ELISA were 70.6 and 95.4%, respectively (8). A threshold OD for the IgG ELISA was 0.145, which resulted in a sensitivity of 93% [confidence interval (CI) = 70–85%] and a specificity of 94% (CI = 85–98%) with an area under the curve (AUC) of >0.9 (Figure 1A).

### Statistical Methods

Categorical variables were compared using χ^2^ or Fisher exact test, and continuous variables were compared using Student *t* testing and Mann-Whitney *U* tests as appropriate. Statistical analysis was performed using Prism (GraphPad, San Diego, CA) and VassarStats (http://faculty.vassar.edu/lowry/VassarStats.html).

## Results

### Demographic Characteristics

A total of 157 graduate medical residents were initially contacted to participate in the study. Of these, 104 residents were eligible by being on inpatient rotations during the study time period. Of those eligible, 44 elected to participate (Supplementary Figure 1). There were no differences between those electing to participate in this study from those who did not, with regard to age or sex. Resident ages ranged from 24 to 36 years. The participants were 64% female. Thirty-one participants (70%) worked on a service or floor that treated COVID-19 patients, with 20 (45%) working in the ED, 5 (11%) in the PICU, 2 (4.5%) on the infectious disease service, and 10 (23%) on the dedicated COVID-19 team. Six participants (14%) worked on more than one COVID-19-exposed team or unit. Each rotation on service lasted ~28 days. ED service rotations consisted of ~16 shifts in a 28-day span. Over the study period, 20 residents reported perceived COVID-19 symptoms, six were tested for SARS-CoV-2 by nasopharyngeal PCR, and one was found to be PCR positive. A summary of the demographic data is presented in Table 1.

**Table 1:** Descriptive characteristics and SARS-CoV-2 seroconversion of study participants separated by SARS-CoV-2 exposure.

	**No. (%)**	
**Characteristic**	**SARS-CoV-2–exposed teams (*n* = 31)**	**Non–SARS-CoV-2 teams (*n* = 13)**
Age, median (range), years	29 (24–34)	29 (27–36)
Female sex	21 (68%)	7 (54%)
Mean postgraduate year, mean (SD), years	2.0 (1.0)	2.5 (1.0)
Seroconversion^c^		
Overall	8 (26%)	4 (31%)
IgM+	4 (13%)	1 (8%)
IgG+	7 (23%)	4 (31%)
Perceived COVID-19 symptoms	16 (52%)	4 (31%)
PCR testing for SARS-CoV-2	3 (10%)^a^	3 (23%)^b^

a *One of three (33%) SARS-CoV-2 PCR tests performed on the graduate medical residents working on the SARS-CoV-2 teams was positive for the virus*.

b *None of three SARS-CoV-2 PCR tests performed on graduate medical residents not working on the SARS-CoV-2 teams was positive for the virus*.

c *Only one pediatric graduate medical resident had a positive IgM result and negative IgG*.

### SARS-CoV-2 Seroconversion

In total, 12 residents tested positive for SARS-CoV-2 antibodies, with 8 of 31 (26%) in the COVID-19 residents and 4 of 13 (31%) of the residents on the non-COVID-19 floors. Eleven of 44 residents (25%) tested positive for SARS-CoV-2 IgG antibodies, and 5 of 44 (11%) tested positive for SARS-CoV-2 IgM antibodies. Of residents on the COVID-19 service, 7 of 31 (23%) tested positive for IgG antibodies, and 4 of 31 (13%) tested positive for IgM antibodies. Of residents on the non-COVID-19 floors, 4 of 13 (31%) tested positive for IgG antibodies, and 1 of 13 (8%) had IgM antibodies (Table 1). Only one resident who seroconverted for IgM had not yet also seroconverted for IgG. There were no differences detected in overall seroconversion (*p* = 0.70), IgG seroconversion rate (*p* = 0.71), or IgM seroconversion rate (*p* > 0.99) between the residents who cared for suspected and confirmed COVID-19 patients and those who did not (Figure 1B). Of the 12 residents with SARS-CoV-2 seroconversion, 7 of 12 (58%) reported no symptoms, and only one tested positive for SARS-CoV-2 by PCR. The median time between reported symptoms and antibody testing was more than 2 months (77.5 days).

## Discussion

**Figure 2:**
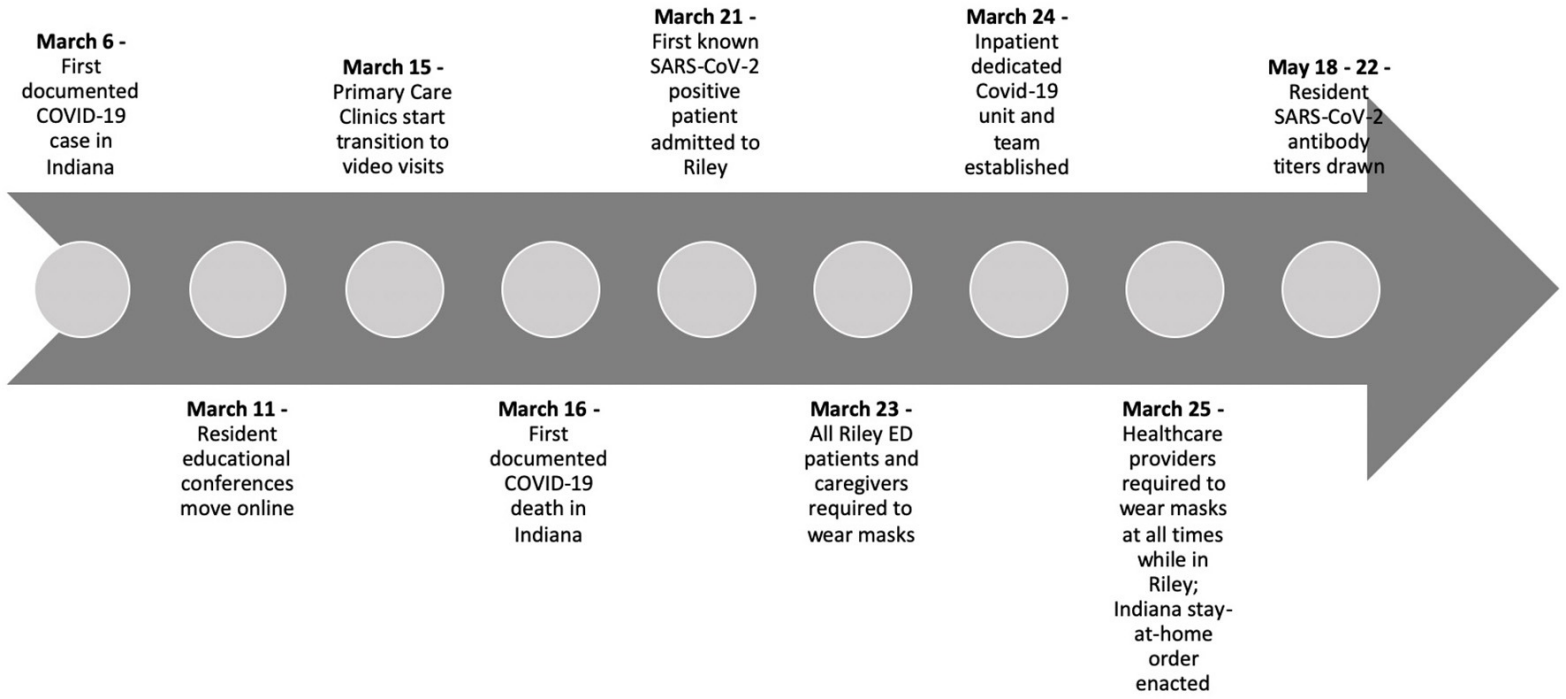
Timeline of Riley children's hospital's response to SARS-CoV-2.

In this cross-sectional study of pediatric graduate medical residents caring for patients in a pediatric tertiary care hospital, one-quarter had antibodies to SARS-CoV-2. We did not find a significant difference in the infection rate between those working on the designated COVID-19 teams and those who did not. Fewer than half of residents who developed IgG also had positive IgM antibodies indicating that the majority of exposures were not acute. Only one resident had a positive IgM result in the absence of IgG seroconversion. A timeline of the COVID-19 pandemic and study is presented in Figure 2 for context.

Our conclusions must be tempered by small sample size as this limited our ability to draw conclusions from these findings. However, our findings further support the importance of PPE, as residents on the SARS-CoV-2-non-exposed teams were just as likely to have seroconversion as those on SARS-CoV-2-exposed team members. These data are in line with the growing evidence supporting the effectiveness of PPE in preventing nosocomial transmission of SARS-CoV-2 (9, 10). Studies in healthcare workers caring for adults have found higher rates of healthcare worker exposure than we describe; however, these studies were performed early in the pandemic prior to stringent PPE guidelines (5, 6).

Pediatric care providers may be at different risk than those caring for adults with COVID-19. Children are susceptible to infection with SARS-CoV-2 infection, but generally present with more mild symptoms than adults; however, severe infections and deaths do still occur in pediatric patients (11, 12). Despite lower incidence of severe disease, children with COVID-19 appear to have high amounts of SARS-CoV-2 viral RNA in their nasopharynx compared to adults (13). Additionally, the rates of transmission from children to adult appear to be lower than adult-to-adult transmission (14). The factors contributing to these changes in transmission dynamics remain unclear. There is still much unknown about SARS-CoV-2 infection in children, including their decreased risk of developing COVID-19, transmission dynamics between children and adults, and the potential role of children as asymptomatic carriers (2, 15–17). Understanding the risk of SARS-CoV-2 transmission from children to adults in the COVID-19 pandemic is vital to help physicians and public health officials direct both infection control procedures and society reopening measures, particularly schools and day-care centers (18). Our findings suggest that creating specific COVID-19 units and teams may not have decreased the risk of SARS-CoV-2 exposure, as it appears that pediatric residents were just as likely to be exposed *via* community spread or other healthcare settings as they were from known hospitalized SARS-CoV-2-positive patients.

There are several limitations to this study. First, our small sample size limited the power to detect true differences between exposure groups. Additionally, our results are limited by the sensitivity and specificity of antibody testing. Our SARS-CoV-2 IgG sensitivity was 93%, so some positive results may not have been detected. Seroconversion progresses over time. Therefore, if the positive control blood samples we used were drawn several months after the PCR validation, sensitivity might be higher. Nonetheless, the SARS-CoV-2 IgG AUC was >0.9, so it represents an “excellent” biomarker with the detection capabilities presented in this study (19). Furthermore, we could not fully control for the heterogeneity of exposure risk on high-risk units, including the ED and intensive care unit; nor could we definitively ensure that the patients admitted to low-risk units were SARS-CoV-2 negative.

Our results suggest that while pediatric graduate medical residents had high rates of antibodies to SARS-CoV-2 as of May 2020, PPE may be effective at limiting transmission within the pediatric hospital setting. This study has limitations, including its lack of longitudinal data and the lag between infection and antibody positivity. Further population-based research on the role of children in transmitting the SARS-CoV-2 virus is needed to allow for a more evidence-based approach toward managing the COVID-19 pandemic.

## Data Availability Statement

The raw data supporting the conclusions of this article will be made available by the authors, without undue reservation.

## Ethics Statement

The studies involving human participants were reviewed and approved by Indiana University Institutional Review Board (IRB). The patients/participants provided their written informed consent to participate in this study.

## Author Contributions

TC and MS conceptualized and designed the study, carried out the initial analysis, drafted the initial manuscript, and reviewed and revised the manuscript. SA, JC, JH, MC, and CP carried out the laboratory studies, and reviewed and revised the manuscript. AS and DH supervised the conceptualization and design of the study and critically reviewed the manuscript for important intellectual content. All authors approved the final manuscript as submitted and agree to be accountable for all aspects of the work.

## Conflict of Interest

The authors declare that the research was conducted in the absence of any commercial or financial relationships that could be construed as a potential conflict of interest.

## Supplementary Material

Supplementary Figure 1 Identification, exclusion, and analysis of residents for this study.
