# Supplementary material for: Placement on COVID-19 Units Does Not Increase Seroconversion Rate of Pediatric Graduate Medical Residents
Source: Front Pediatr. 2021 Apr 29;9:633082. doi: 10.3389/fped.2021.633082 (PMC8116566; doi:10.3389/fped.2021.633082)
Supplement: Supplementary file 1 [file Data_Sheet_1.docx]

**Supplemental Material: Resident Survey**

Age: _______

Post-Graduate Year: _____

Gender:

Female

Male

Did you work on any of the following teams from February 1, 2020 to May 22, 02020?

Emergency Department

Pediatric Intensive Care Unit

Infectious Disease Service

COVID Pediatric hospital medicine team

Do you believe you have had an illness since February 1st which was likely to have been COVID-19?

Yes

No

What symptoms did you experience?

Fever

Headache

Malaise

Respiratory Symptoms

GI Symptoms

Myalgias

Anosmia (loss of smell)

Other: ______________

Did you have a COVID test performed?

Yes

No

If yes, what was the result of your COVID test?

Positive

Negative

Equivocal

Don’t know
