# Supplementary material for: Placement on COVID-19 Units Does Not Increase Seroconversion Rate of Pediatric Graduate Medical Residents
Source: Front Pediatr. 2021 Apr 29;9:633082. doi: 10.3389/fped.2021.633082 (PMC8116566; doi:10.3389/fped.2021.633082)
Supplement: Supplementary Figure 1 — Identification, exclusion, and analysis of residents for this study. [file Data_Sheet_2.docx]

## Supplemental Figure 1. Identification, exclusion and analysis of residents for this study.

Declined Participation or No Blood Draw

N = 60

Excluded from study (Not inpatient)

N = 43

All Graduate Medical Residents

N = 157

Eligible for inclusion (inpatient rotation March – May 2020)

N = 104

Included in Analysis

N = 44
